# Gut microbiome differences by serostatus in rheumatoid arthritis: a systematic review

**DOI:** 10.3389/fimmu.2026.1722255

**Published:** 2026-03-25

**Authors:** Madiyar Nurgaziyev, Samat Kozhakhmetov, Argul Issilbayeva, Zharkyn Jarmukhanov, Ayaulym Nurgaziyeva, Shynggys Sergazy, Malika Kuantkhan, Laura Chulenbayeva, Almagul Kushugulova

**Affiliations:** Laboratory of Microbiome, Center for Life Sciences, National Laboratory Astana, Nazarbayev University, Astana, Kazakhstan

**Keywords:** ACPA, gut microbiome, RF, rheumatoid arthritis, serological status, systematic review

## Abstract

**Background:**

Rheumatoid arthritis (RA) is a heterogeneous autoimmune disease in which serological status, defined by rheumatoid factor (RF) and anti-citrullinated protein antibodies (ACPA), influences disease course. Alterations of the gut microbiome have been implicated in RA, but differences between seropositive and seronegative patients, and between seropositive RA and healthy controls, have not been systematically reviewed.

**Methods:**

PubMed, Scopus, Web of Science, and the Cochrane Library were searched to July 2025 for observational studies of adult RA patients reporting RF and/or ACPA status with gut microbiome analysis. Two reviewers independently screened, extracted data, and assessed quality using the Newcastle–Ottawa Scale (NOS).

**Results:**

Eight studies published between 2016 and 2024 met the inclusion criteria. Six investigated both RF and ACPA, while two focused primarily on ACPA. In seropositive RA, higher abundances of *Collinsella* and *Blautia* and lower levels of *Faecalibacterium* were consistently reported. Several studies demonstrated reduced α-diversity in seropositive patients compared with seronegative RA or healthy controls, particularly in preclinical or early disease, while established RA showed no consistent differences. Findings for β-diversity were heterogeneous, with some cohorts reporting significant associations with serostatus, whereas others found no clear separation.

**Conclusions:**

Seropositive RA, especially ACPA-positive, is frequently associated with reduced microbial diversity and distinct compositional shifts compared with seronegative RA and healthy controls. Larger standardized studies are required to validate these associations and assess their biomarker potential.

**Systematic review registration:**

https://www.crd.york.ac.uk/prospero/, identifier CRD420251140715.

## 1 Introduction

Rheumatoid arthritis (RA) is a chronic autoimmune disease that primarily affects the joints, leading to persistent inflammation, cartilage destruction, and disability. The global prevalence of RA is estimated at around 0.5–1%, with women and individuals in middle age being more frequently affected (1, 2). Besides joint involvement, RA is also linked to comorbidities such as cardiovascular disease and metabolic complications, which contribute to reduced quality of life and increased mortality (1, 3). Despite important progress in disease-modifying antirheumatic drugs (DMARDs) and biologics, the exact mechanisms that drive differences in disease onset, clinical presentation, and outcomes remain only partly understood (4). This makes it important to explore biological and environmental factors that may explain disease heterogeneity.

Serological status is one of the main features that separates RA subgroups. Rheumatoid factor (RF) and anti-citrullinated protein antibodies (ACPA) are the two most studied markers. RF is an autoantibody directed against the Fc portion of IgG and is detected in approximately 60–80% of RA patients, but it can also be present in other autoimmune or inflammatory conditions, limiting its disease specificity (5, 6). In contrast, ACPA exhibit a high specificity for RA, exceeding 90–95% (7), and can appear years before the clinical onset, serving as predictors for disease development (8, 9). Both RF and ACPA positivity are generally associated with more severe disease, faster joint damage, and a higher risk of extra-articular manifestations (10, 11). In contrast, seronegative RA often shows milder disease activity, but it is clinically diverse and sometimes difficult to distinguish from other inflammatory joint conditions (12). Therefore, serological markers are not only important for classification, but may also point to different pathogenic mechanisms.

In recent years, the gut microbiome has been recognized as an important factor in shaping immune responses and influencing the development of autoimmune diseases. Alterations in microbial composition, often referred to as dysbiosis, have been linked to loss of immune tolerance, systemic inflammation, and disease progression in conditions such as inflammatory bowel disease, multiple sclerosis, and type 1 diabetes (13, 14). Similar observations have been made in RA, where patients frequently show reduced microbial diversity and an increased abundance of specific taxa such as *Prevotella copri*, *Collinsella aerofaciens*, and *Eggerthella lenta* (15–17). These microbes have been associated with enhanced Th17 responses, intestinal barrier dysfunction, and altered metabolic pathways that may contribute to joint inflammation (18). However, the exact relationship between microbiome alterations and different RA phenotypes, including seropositive and seronegative disease, is still not fully understood.

Although increasing evidence supports the role of the gut microbiome in RA, few studies have systematically evaluated how microbial alterations differ according to serological status. Most existing research focuses on overall comparisons between RA patients and healthy controls, while subgroup analyses of seropositive versus seronegative disease are often underrepresented or only reported as secondary findings (16, 17, 19).

To date, no systematic review has synthesized the available evidence specifically addressing gut microbiome alterations in relation to RA serostatus. Understanding whether distinct microbial profiles are associated with seropositive versus seronegative disease is important, as it may reflect different pathogenic pathways, refine disease classification, and identify potential biomarkers or therapeutic targets. Therefore, the aim of this review is to summarize and critically evaluate current evidence on gut microbiome composition stratified by serological status in RA, and to highlight potential mechanisms linking serostatus with host–microbiome interactions.

## 2 Materials and methods

### 2.1 PICO question

We aimed to address the following research question: Do adult patients with RA (P), categorized by serological status (RF and/or ACPA), (I) show differences in gut microbiome composition (O) compared to seronegative RA patients or healthy controls (C)? In other words, our focus was on whether seropositivity (RF and/or ACPA) is associated with distinct gut microbial profiles. The PICO framework was defined as follows: Population - adult patients diagnosed with RA; Intervention/Exposure - seropositive status (RF and/or ACPA positive); Comparison - seronegative RA patients (RF and/or ACPA negative) or healthy controls; Outcome - gut microbiome composition, microbial metabolites, and related immune mechanisms.

### 2.2 Inclusion and exclusion criteria

Studies were eligible if they met the following criteria (1): original human research; (2) published in English between 2013 and 2025; (3) included RA patients diagnosed according to ACR/EULAR criteria; (4) reported serological status (RF and/or ACPA); (5) investigated gut microbiome composition; (6) used observational study designs (cohort, case–control, or cross-sectional); and (7) were published in peer-reviewed journals.

We excluded (1) animal or *in vitro* studies; (2) articles in languages other than English; (3) reviews, case reports, editorials, or conference abstracts; (4) studies without RF/ACPA stratification; and (5) studies not reporting microbiome-related outcomes.

### 2.3 Search strategy

A systematic search was performed in PubMed, Scopus, Web of Science, and the Cochrane Library up to July 23, 2025. We combined controlled vocabulary and free-text terms for rheumatoid arthritis, the gut microbiome, and serological markers. Search strings included combinations such as “rheumatoid arthritis” OR “RA” AND (“gut microbiome” OR “gut microbiota” OR “intestinal microbiota” OR “gut flora” OR “intestinal flora” OR “dysbiosis”) AND (“rheumatoid factor” OR “RF” OR “anti-cyclic citrullinated peptide” OR “ACPA” OR “anti-CCP” OR “serostatus” OR “serological status” OR “autoantibodies”). In addition, we manually screened reference lists of relevant articles and previous reviews to identify further eligible studies. The complete search strategies for each database are detailed in **Supplementary Table 1**.

### 2.4 Study selection and data extraction

Two reviewers independently screened titles and abstracts andxassessed full texts against the eligibility criteria. Any disagreements were resolved by discussion or, if needed, consultation with a third reviewer. Reporting followed PRISMA recommendations (**Supplementary Table 2**). Data were extracted using a standardized form, including study characteristics (author, year, country, design, sample size, population details, serological status, microbiome assessment methods, and main findings) (20). The protocol of this systematic review was registered on PROSPERO with ID CRD420251140715. Heatmap visualization was generated using Python (version 3.13.9) within the Anaconda distribution, employing the seaborn and matplotlib libraries.

### 2.5 Quality assessment

The risk of bias of the included studies was assessed using the Newcastle–Ottawa Scale (NOS), a widely used tool for observational research (21, 22). For this review, the NOS was adapted for cross-sectional and case–control designs and applied across three domains. The Selection domain (maximum of 4 points) assessed whether studies used a clear case definition, evaluated representativeness of the sample, and ensured appropriate selection and definition of controls. The Comparability domain (maximum of 2 points) reflected the extent to which potential confounders such as age, sex, BMI, smoking, or treatment were considered, either by adjustment or by matching. The Exposure/Outcome domain (maximum of 3points) evaluated whether microbiome assessment was clearly described, whether the same methods were used for cases and controls, and whether non-response or missing data were adequately reported. Following thresholds applied in previous systematic reviews (21, 23), scores of ≤5 were regarded as low quality, 6–7 as moderate, and 8–9 as high quality. The appraisal was conducted independently by two reviewers, with disagreements resolved by consensus or, when necessary, by a third reviewer.

## 3 Results

### 3.1 Study screening and selection

**Figure 1:**
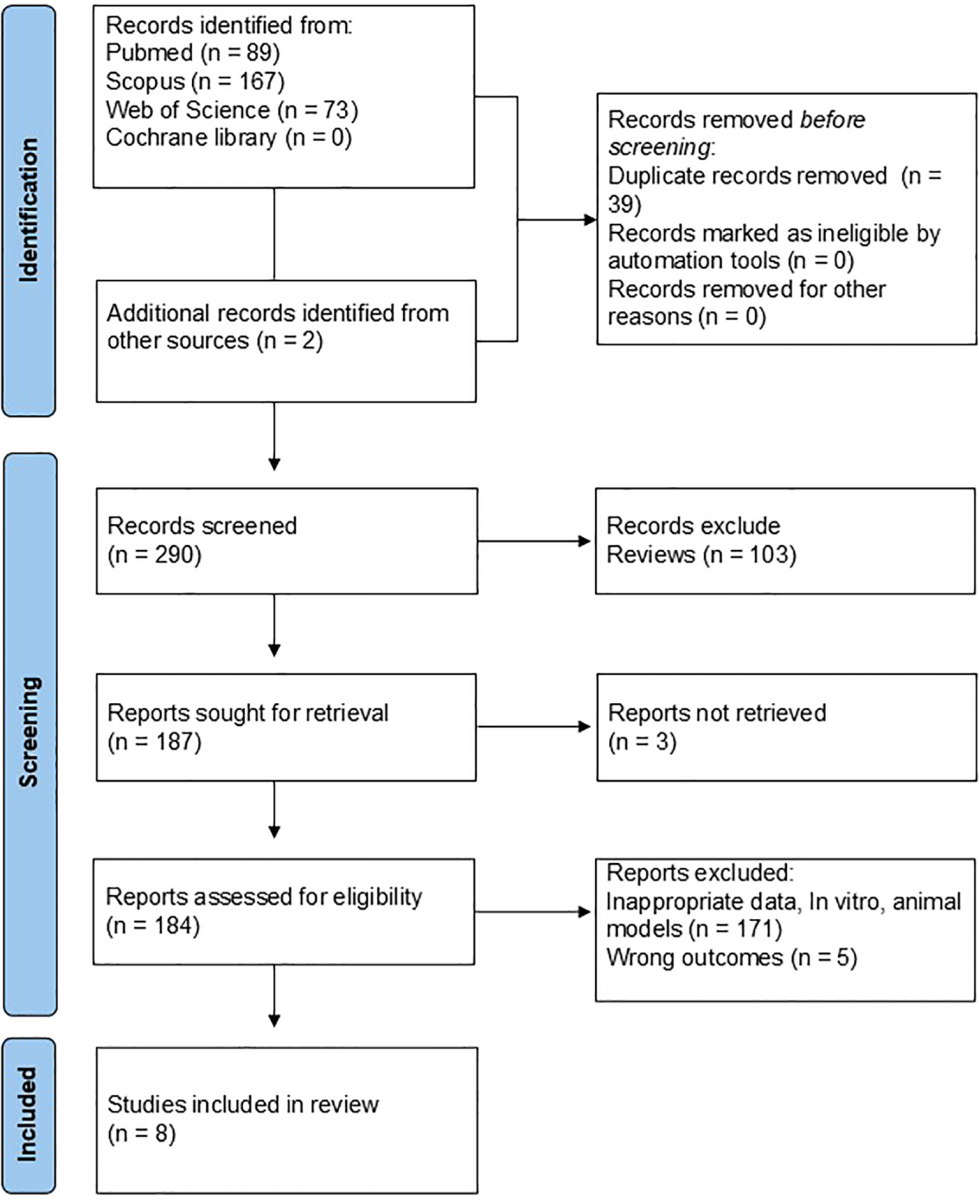
Flowchart detailing the search methods and findings according to PRISMA guidelines.

The database search identified 329 records in total, including 89 from PubMed, 167 from Scopus, 73 from Web of Science, and none from the Cochrane Library. In addition, two records were found through manual searches of reference lists. After removing 39 duplicates, 290 unique records were screened. Of these, 103 were excluded at the title and abstract level because they were reviews. Full texts of 187 articles were obtained for detailed assessment, of which three could not be accessed. Among the 184 studies assessed for eligibility, 171 were excluded due to inappropriate data, including *in vitro* or animal studies and studies that did not analyze gut microbiome outcomes. An additional five studies were excluded because they did not provide information on rheumatoid arthritis (RA) serostatus. In the end, eight studies met the predefined inclusion criteria and were included in this systematic review (**Figure 1**).

### 3.2 Overview of included studies

Studies published between 2016 and 2024 met the eligibility criteria and were included in this review (**Table 1**). These studies were conducted across Asia, Europe, and North America, with sample sizes ranging from fewer than 50 participants to nearly 200. Most were cross-sectional case–control studies. One study also examined baseline microbiome profiles in relation to treatment response.

**Table 1:** Characteristics and main findings of the included studies.

Authors, year (country/ region)	Study design	Study sample	Serostatus (RF/ACPA)	Disease treatment	Microbiome method	Key outcomes	Study quality (NOS)
Chen et al., 2016 (China) (16)	Observational case–control study	40 RA, 15 FDR, 17 HC (female RA- 70% female HC-81% male RA- 30% male HC- 19%)	RF+ 100%; ACPA+ 83%	Methotrexate (MTX), hydroxychloroquine, and prednisone in some patients	16S rRNA (Illumina MiSeq; V3–V5)	***Composition:*** **RF and ACPA positive vs HC:** *↑ Eggerthella, Collinsella;* *↓Faecalibacterium;* ***Diversity:*** **RF levels ↑ →** α-diversity **↓ (**p < 0.1) β-diversity associated with ↑RF, ↑CRP, ↑disease duration, MTX/HCQ treatment. **RA vs HC** significant β-diversity (p < 0.001)	7 (Moderate)
Chiang et al., 2019 (Taiwan) (26)	Cross-sectional case–control study	128 RA, 20 HC (female RA- 79.7% female HC-80% male RA- 20.3% male HC- 20%)	RF+ 75.8%; ACPA+ 73.4%	csDMARDs; corticosteroids; non-steroidal anti-inflammatory drugs	16S rRNA (Illumina MiSeq; V1–V3)	***Composition:*** **RF-positive vs RF-negative:** ↑ *Blautia*, *Collinsella* **ACPA-positive vs ACPA-negative:** ↑ *Blautia*, *Akkermansia*, *Clostridiales* ***Diversity:*** **RF-negative vs HC:** ↓ α-diversity (p < 0.05) **ACPA-positive vs HC:** ↓ α-diversity (p = 0.053) **Seropositive vs Seronegative**: ↓ α -diversity (p < 0.05) no significant β-diversity difference	7 (Moderate)
Sun et al., 2019 (China) (28)	Case–control, single-center	66 RA/60 HC (female RA- 77.3% female HC-51.7% male RA- 22.7% male HC- 48.3%)	RF+ 30.3%; ACPA + 37.9%	NR	16S rRNA (Illumina MiSeq; V1–V3)	***Composition:*** **RF-positive:** ↑ Dorea, Alloprevotella; ↓ Sutterella, Megamonas, Faecalibacterium **ACPA-positive:** ↑ Dorea, Alloprevotella ***Diversity:*** **RA vs HC:** ↓ α-diversity (p < 0.05); significant difference in β-diversity (no serostatus analysis)	6 (Moderate)
Mena-Vázquez et al., 2020 (Spain) (27)	Cross-sectional case–control study	40 RA, 40 HC (female RA- 75% female HC-75% male RA- 25% male HC- 25%)	RF+ 80%; ACPA+ 70%	DMARDs, biologic therapy	16S rRNA (V2–4–8 & V3–6, Ion Torrent S5)	***Composition:*** **ACPA-positive:** ↑ Collinsella aerofaciens associated with high ACPA titers ***Diversity:*** **RA vs HC:** no significant difference in α-diversity **RA vs HC:** β-diversity trend only (ANOSIM test, p = 0.07) (no serostatus analysis)	7 (Moderate)
Luo et al., 2023 (USA) (24)	Cross-sectional case–control study	53 Pre-RA (ACPA+)/38 HC (female Pre-RA- 52.8% female HC-63.2% male RA- 47.2% male HC- 36.8%)	RF+ 24.5%; ACPA + 100%	No DMARDs/biologics, no regular NSAIDs	16S rRNA (Illumina MiSeq; V3–V4)	***Composition:*** **ACPA-positive Pre-RA vs HC:** ↑ Lactobacillus, Raoultibacter, Eubacterium_brachy_group, Enorma, Holdemania; ↓ Ruminococcus_2, Pseudomonas, Ruminiclostridium_5, Coprococcus_2, Ruminococcaceae_UCG-009; ***Diversity:*** **ACPA-positive Pre-RA vs HC:** ↓ α-diversity	8 (High)
Koh et al., 2023 (South Korea) (29)	Cross-sectional case–control study	94 RA/30 HC (female RA- 92.6% female HC-100% male RA- 7.4% male HC- 0%)	RF+ 76.3%; ACPA+ 84.3%	csDMARDs and/or bDMARDs; glucocorticoids and NSAIDs	16S rRNA (Illumina MiSeq; V3–V4	**Diversity:** **ACPA/RF-positive:** no significant α/β-diversity changes	6 (Moderate)
Yun et al., 2023 (China) (30)	Cross-sectional case–control study	40 RA/7 HC (female only)	NR	No probiotics, antibiotics, immunosuppressants, or biologics for ≥1 month	16S rRNA (V3–V4, Illumina NovaSeq)	**RF-positive:** ↑ Megamonas, Dialister, SMB53; **ACPA-positive:** ↑ Blautia **Diversity:** **RA vs HC:** β-diversity differed significantly (no serostatus analysis)	5 (Low)
Nurgaziyev et al., 2024 (Kazakhstan) (25)	Cross-sectional case–control study	77 RA/113 HC (female only)	RF+ 77.9%, ACPA + 54.5%	DMARDs, biologic therapy, NSAIDs	16S rRNA (V1–V3, Illumina NovaSeq)	**RF-positive:** ↑ *Eubacterium ruminantium*, *Monoglobus*; **RF-negative:** ↑ *Prevotella*, *Colidextribacter*, *Butyricimonas*; **ACPA-positive:** ↑ *g_UCG_005*, *Odoribacter*, *Ligilactobacillus*; **ACPA-negative:** ↑ *Faecalibacterium*, *Haemophilus*. **Diversity:** no significant α/β-diversity differences by RF/ACPA status.	8 (High)

RA, rheumatoid arthritis; HC, healthy controls; FDR, first-degree relatives; RF, rheumatoid factor; ACPA, anti-citrullinated protein antibodies; bDMARDs, biologic DMARDs; csDMARDs, conventional synthetic DMARDs; NR, not reported.

Bold values indicate key values highlighted for clarity.

Six studies reported both RF and ACPA, while others focused only on one marker (ACPA). Two studies concentrated on ACPA-positive patients, one mainly investigated RF, and one included ACPA-positive individuals at preclinical high risk of RA, allowing evaluation of microbial alterations before disease onset. All studies used 16S rRNA gene sequencing, although sequencing platforms and targeted variable regions differed; two extended beyond taxonomic profiling by integrating metabolomic data or performing predictive functional pathway analysis. Reporting of treatment exposure varied in the articles. Some studies excluded participants with recent use of antibiotics, probiotics, or DMARDs, while others included patients receiving conventional or biologic DMARDs, glucocorticoids, or NSAIDs.

Although all studies were observational and primarily cross-sectional case–control in design, they differed in patient populations, comparison groups, and analytical approaches. Most studies compared RA patients with healthy controls. One study included first-degree relatives, and another examined ACPA-positive preclinical individuals. Several studies also incorporated metabolomic or functional pathway analyses or combined human data with experimental models. Across cohorts, the presence of autoantibodies (RF and/or ACPA) was consistently associated with distinct microbial features.

### 3.3 Quality assessment of included studies

Total NOS scores of the eight included studies ranged from 5 to 8 out of 9, indicating that most were of moderate to high quality (**Table 2**). Two studies (24, 25) achieved the highest score of 8 points and were rated as high quality. Four studies (16, 26–28) scored 6–7 points and were considered of moderate quality. One study (28, 29) also fell into the moderate category with a total of 6 points. Only one study (30) was rated as low quality, with a total score of 5.

**Table 2:** NOS assessment of included studies.

First author & publication year	Selection				Comparability		Exposure			
	Is the case definition adequate?	Representativeness of the cases	Selection of controls	Definition of controls	Comparability of baseline characteristic 1 (sex)	Comparability of baseline characteristic 2 (age, smoking, medications)	Ascertainment of exposure	Same method of ascertainment for cases and controls	Non-response rate	Total
Chen et al., 2016 (16)	1	1	1	1	1	0	1	1	NR	7
Chiang HI., 2019 (26)	1	1	1	1	1	1	1	0	NR	7
Sun Y., 2019 (28)	1	1	1	0	1	0	1	0	NR	6
Mena-Vázquez N 2020 (27)	1	1	1	1	1	1	1	0	NR	7
Luo et al., 2023 (24)	1	1	1	1	1	1	1	1	NR	8
Koh et al., 2023 (29)	1	1	1	0	1	0	1	1	NR	6
Yun et al., 2023 (30)	1	0	1	1	1	0	1	0	NR	5
Nurgaziyev et al., 2024 (25)	1	1	1	1	1	1	1	1	NR	8

### 3.4 Gut microbiome composition according to serostatus

**Figure 2:**
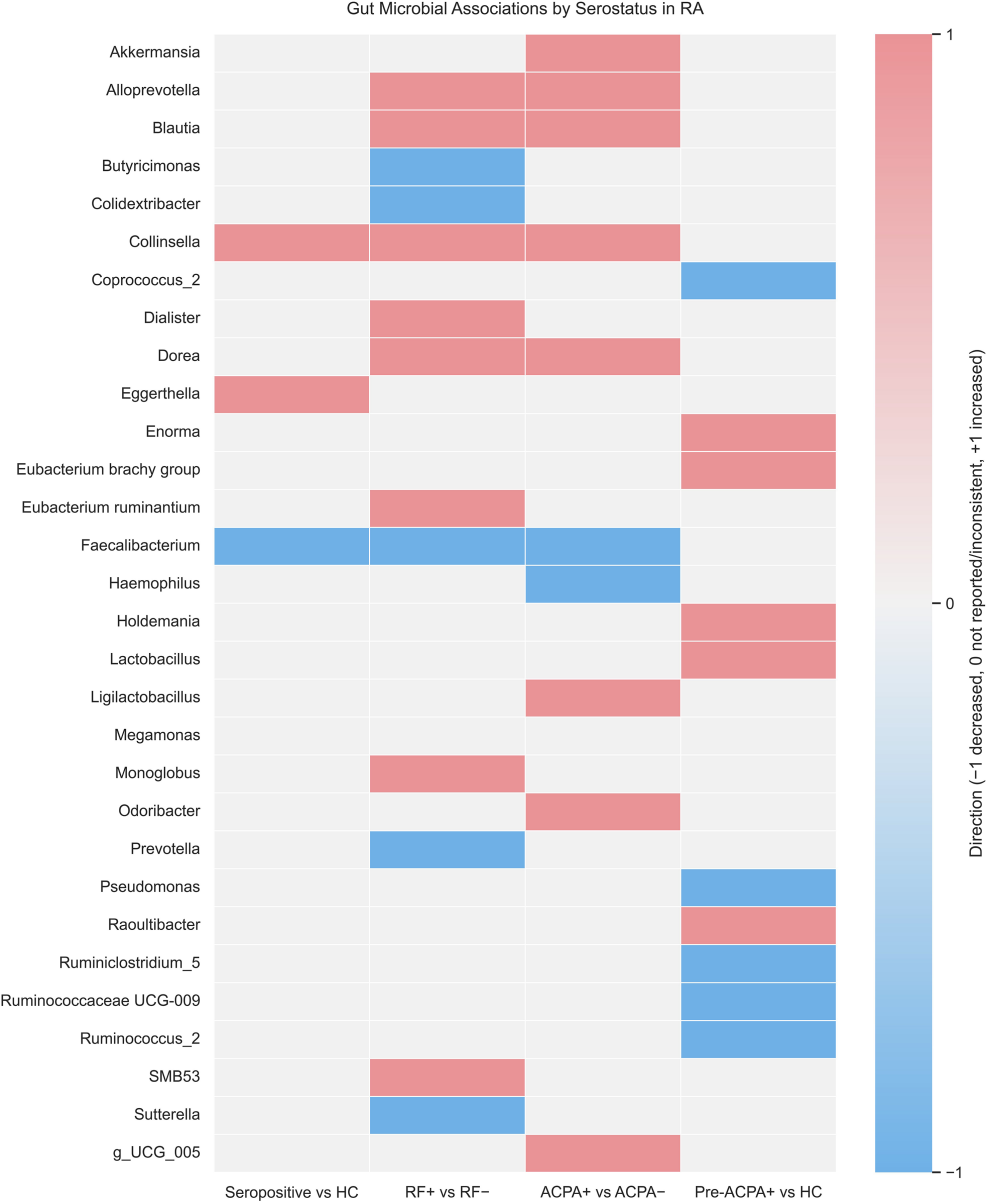
Heatmap summarizing direction of reported associations between gut microbial taxa and serological status in rheumatoid arthritis. Red indicates increased abundance; blue indicates decreased abundance; blank cells indicate not reported. Values represent qualitative direction only. Taxa with inconsistent direction across studies (Megamonas) are shown as neutral (0).

Differences in taxonomic composition according to RF and ACPA were reported in several studies. The direction of reported microbial associations is summarized in **Figure 2**. Chen et al. (16) showed enrichment of *Eggerthella* and *Collinsella* and depletion of *Faecalibacterium* in seropositive patients compared with controls. In the study by Chiang et al. (26), RF-positive patients had higher abundances of *Blautia* and *Collinsella*, while ACPA-positive patients were enriched in *Blautia, Akkermansia*, and members of the *Clostridiales* compared with ACPA-negative cases. Sun et al. (28) reported that *Dorea* and *Alloprevotella* correlated positively with RF and ACPA, whereas *Sutterella, Megamonas*, and *Faecalibacterium* were negatively correlated with RF levels. Mena-Vázquez et al. (27) identified *Collinsella* as positively associated with high ACPA titers.

In the preclinical setting, Luo et al. (24) found that ACPA-positive individuals were enriched in *Lactobacillus, Raoultibacter, Eubacterium brachy group*, *Enorma*, and *Holdemania*, and depleted in *Ruminococcus_2, Pseudomonas, Ruminiclostridium_5, Coprococcus_2, and Ruminococcaceae UCG-009*. Yun et al. (30) described higher *Megamonas, Dialister*, and *SMB53* in RF-positive patients and increased *Blautia* in ACPA-positive cases. Nurgaziyev et al. (25) reported that *Eubacterium ruminantium* and *Monoglobus* were linked with RF-positive disease, while *Prevotella, Colidextribacter*, and *Butyricimonas* were enriched in RF-negative patients. In relation to ACPA*, g_UCG_005, Odoribacter, and Ligilactobacillus* were associated with ACPA-positive cases, whereas *Faecalibacterium* and *Haemophilus* were more abundant in ACPA-negative RA.

In contrast to these studies, Koh et al. (29) did not analyze gut microbiome composition according to RF or ACPA status.

### 3.5 Gut microbiome diversity according to serostatus

Seven of the included studies assessed α-diversity and seven examined β-diversity (**Table 1**). Only part of them analyzed differences according to RF or ACPA.

Chen et al. (16) reported that higher RF levels and longer disease duration were both associated with reduced species richness, and elevated RF was also linked to lower Shannon diversity (p < 0.05). Chiang et al. (26) found significantly lower α-diversity in RF-negative patients compared with healthy controls (p < 0.05) and a borderline decrease in ACPA-positive cases (p = 0.053). When RF- and ACPA-positive patients were combined, the seropositive group had reduced α-diversity diversity compared with controls (p < 0.01), and within RA patients, seropositives showed lower α-diversity than seronegatives (p < 0.05). Luo et al. (24) reported that ACPA-positive individuals at the preclinical stage had reduced α-diversity diversity compared with healthy controls (p < 0.05). By contrast, Koh et al. (29) and Nurgaziyev et al. (25) did not observe significant differences in α-diversity between seropositive and seronegative RA. Associations between β-diversity and serological status were examined in several studies. Chen et al. (16) found that community structure was significantly influenced by rheumatoid factor levels, as well as disease duration, CRP levels, and treatment with MTX or hydroxychloroquine. In the study by Chiang et al. (26), no significant differences were observed in β-diversity when patients were grouped by RF or ACPA status. Similarly, Koh et al. (29) and Nurgaziyev et al. (25) did not detect differences in community clustering between seropositive and seronegative RA patients. By contrast, Luo et al. (24) reported that ACPA-positive individuals at the preclinical stage showed a distinct β-diversity profile compared with healthy controls.

Other studies assessed RA patients against healthy controls without analyzing RF or ACPA status. Sun et al. (28) and Yun et al. (30) both identified significant β-diversity differences between RA and controls, while Mena-Vázquez et al. (27) reported only a non-significant trend (ANOSIM p = 0.07).

## 4 Discussion

Our review shows that serological status in RA is linked to characteristic changes in the gut microbiome. Across several studies, seropositive disease, particularly in ACPA-positive patients, was associated with higher levels of genera such as *Collinsella* and *Blautia* (16, 26, 27, 30), together with a consistent reduction in *Faecalibacterium* (16, 28), a known butyrate producer (31). Other observations included positive associations of RF with *Dorea* and *Alloprevotella* (28) and of ACPA with *Odoribacter* and *Ligilactobacillus* (25). In contrast, *Prevotella*, *Faecalibacterium*, and *Haemophilus* appeared in seronegative RA, although this was only reported in individual cohorts. Importantly, evidence from preclinical ACPA-positive individuals pointed to distinct microbial profiles, including enrichment of *Lactobacillus* and *Raoultibacter* and depletion of *Ruminococcus* and *Pseudomonas*, suggesting that microbial alterations may precede clinical onset (24). Preclinical cohorts were included in this review because they provide unique understanding of the temporal relationship between autoantibody status and gut microbiome changes. Unlike patients with established RA, individuals who are ACPA-positive but have not yet developed arthritis allow us to examine whether microbial alterations occur before joint inflammation becomes clinically evident.

From an immunological perspective, the taxa most consistently associated with seropositive RA are biologically relevant to mucosal immune regulation. *Collinsella* has been shown to increase gut epithelial permeability and induce expression of pro-inflammatory cytokines such as IL-17A, a key mediator of Th17 responses, and to exacerbate arthritis in murine models. These observations suggest that expansion of *Collinsella* in RA may be linked to altered barrier integrity and pro-inflammatory immune signaling (32, 33). In comparison, *Faecalibacterium*, a major butyrate-producing genus, plays a key immunoregulatory role by supporting epithelial integrity and promoting anti-inflammatory responses, including maintenance of Th17/Treg balance (34). A couple of studies included in this review reported depletion of *Faecalibacterium* in seropositive RA cohorts (13, 25), and given its established immunoregulatory functions, such depletion may reflect reduced availability of immunoregulatory microbial metabolites and impaired mucosal tolerance (35). *Blautia* represents a functionally heterogeneous genus, and its enrichment in seropositive RA may reflect disease-associated microbial restructuring rather than a consistently protective effect. Supporting this context dependency, increased abundance of *Blautia* has been associated with reduced levels of key immune cell populations, including CD4^+^ T cells and regulatory T cells, in RA patients, suggesting that genus-level enrichment should be interpreted cautiously and may not necessarily correspond to immunological benefit (36).

One important limitation of the current evidence relates to the taxonomic resolution of microbiome analyses. Most included studies relied on 16S rRNA gene sequencing, which generally enables classification at the genus level but does not capture species or strain-level heterogeneity. Increasing evidence indicates that different species within the same genus may influence immune function differently, and that strain-level variation within a single species can result in distinct functional properties (37). Moreover, data suggest that strains colonizing patients with rheumatoid arthritis may differ functionally from those present in healthy individuals (38). This has been particularly well documented for *Prevotella copri*, in which distinct strains have been associated with divergent immune and metabolic profiles (39, 40), and more broadly for metabolically important commensals such as *Eubacterium rectale*, which also exhibit marked strain-dependent functional variability (38, 41). These observations highlight that genus level associations should be interpreted with caution and underscore the need for future studies employing higher resolution approaches, including shotgun metagenomics, strain-resolved analyses, and functional profiling.

Measures of diversity support these findings but also highlight the variability across studies. Several cohorts reported reduced α-diversity in seropositive patients compared with seronegative RA or healthy controls, particularly in preclinical and early RA (16, 24, 26). By contrast, studies in established RA often failed to show such differences (25, 29). The situation was similar for β-diversity: some analyses linked autoantibody status with overall microbial community structure (16, 24), whereas others found no significant separation by RF or ACPA (26, 29).

Previous review has investigated the relationship between the gut microbiome and RA, but without focusing on serological status. The study demonstrated an expansion of *Prevotella copri* in new-onset or untreated RA, suggesting a potential role in disease susceptibility (18). In our review, associations with *Prevotella* were less consistent and not strongly linked to autoantibody-positive disease. This suggests that *Prevotella* may reflect environmental exposures (39) or early dysbiosis rather than being specifically tied to seropositivity. Others reported reductions in butyrate-producing taxa such as *Faecalibacterium prausnitzii* and members of the *Clostridiales*, which are of particular relevance to RA pathogenesis. These commensals normally maintain epithelial barrier integrity and promote anti-inflammatory immune responses through regulatory T cell induction. Their depletion leads to reduced butyrate availability, impaired barrier function, and enhanced pro-inflammatory Th17 activity, mechanisms that may facilitate systemic immune activation (34, 42) and contribute to the development of RA. In accordance with this, several studies included in our review reported reduced abundance of *Faecalibacterium* in seropositive patients, suggesting that loss of these protective taxa may be linked to autoantibody production and early immunological dysregulation. These mechanistic perspectives may also carry clinical implications. The consistent association between autoantibody positivity and distinct gut microbial features has potential clinical relevance. If validated in larger cohorts, specific taxa such as *Collinsella, Eggerthella*, or the depletion of *Faecalibacterium* could serve as biomarkers to identify individuals at higher risk of developing RA or to differentiate seropositive from seronegative disease (16, 26, 27, 43). Such microbial signatures might also provide prognostic information, for example by predicting disease progression in preclinical ACPA-positive individuals (24). The clinical significance of these findings lies in their potential to inform early disease interception. Microbiome alterations detected in ACPA-positive individuals prior to clinical onset suggest that gut microbial features may help identify individuals at increased risk of progression to RA. In practical terms, microbiome profiling could be considered as an additional layer of risk assessment alongside established serological markers, helping to identify individuals who may benefit from closer follow-up or early preventive approaches. This supports the concept that microbiome-informed strategies may be most relevant before irreversible joint damage occurs.

Recent systematic reviews have also emphasized that variability across studies may result from differences in disease stage, treatment exposure, and geographic background. For example, Bodkhe et al. (19) demonstrated that microbial alterations in RA were partly normalized after DMARD therapy, underscoring the confounding effect of treatment on microbiome composition. Similarly, Chu et al. (23) highlighted that inconsistencies in reported taxa often reflect heterogeneous methodologies and populations, rather than the absence of a true signal (44). Also, Gupta et al. (45) pointed out that treatment status and clinical factors can substantially modulate microbial diversity, which may conceal signals in established RA. Our analysis extends these observations by showing that serological status itself may be an additional source of heterogeneity, with dysbiotic features more consistently observed in autoantibody-positive patients. This suggests that division by RF and ACPA is important for future microbiome studies in RA to reduce variability and to refine associations with clinical outcomes.Taken together, these findings highlight not only the methodological and biological factors that shape microbiome research in RA but also their potential translational relevance. Beyond diagnostics, the gut microbiome is increasingly recognized as a therapeutic target (46). Several reviews have proposed that modulation of microbial communities through diet, probiotics, or fecal microbiota transplantation may complement disease-modifying therapies and help restore immune balance (47–49). These strategies may be most effective if applied in the earliest stages of disease, before irreversible joint damage occurs. Another clinical implication of our findings is the potential to refine patient classification by serostatus and management strategies. Seropositive RA is typically associated with more aggressive disease and a greater frequency of extra-articular manifestations (10, 11), and the consistent link between autoantibody status and gut dysbiosis suggests that microbial features could further sharpen risk profiling. Incorporating microbiome data into current clinical practice protocols may help identify which patients are most likely to benefit from early intensive therapy, while also guiding the design of tailored lifestyle or dietary interventions (50, 51). In this way, the integration of serological and microbial markers could support a more personalized approach to RA treatment.

The main strength of this systematic review is its novel focus on gut microbiome alterations according to serological status in RA, an aspect that has not been systematically addressed in previous reviews. By synthesizing data from both established and preclinical cohorts, we were able to highlight differences between seropositive and seronegative disease and demonstrate that dysbiosis is more consistently observed in autoantibody positive patients. The review also followed a structured methodology, including a comprehensive literature search across multiple databases, independent screening and data extraction by two reviewers, and quality assessment using the Newcastle-Ottawa Scale. Reporting was aligned with PRISMA 2020 guidelines (20), and the protocol was prospectively registered in PROSPERO, further strengthening the transparency and reproducibility of our approach.At the same time, several limitations need to be considered. The number of eligible studies was small, and most involved small sample sizes, limiting the robustness and generalizability of the findings. Considerable heterogeneity in study design, bioinformatics pipelines, and patient populations complicates direct comparisons, even though sequencing methods were largely similar across studies. Treatment exposure was variably reported and rarely controlled for, despite evidence that DMARDs influence the gut microbiome. Furthermore, many studies compared seropositive patients only with healthy controls rather than directly with seronegative RA, and some reported associations for RF or ACPA alone instead of both markers. This lack of consistent separation complicated analysis and restricted our ability to identify microbial features specific to serological subsets.

Sex-related differences may represent an additional source of heterogeneity in studies of the gut microbiome in RA, as RA is strongly female-predominant and sex hormones influence immune regulation and gut microbial composition (52). However, most studies included in this review were female-dominant or female-only and did not perform sex-stratified analyses according to RF or ACPA status. Therefore, the interaction between sex, serological status, and gut microbiome composition could not be systematically assessed and remains an important direction for future research.

Finally, most included studies were cross-sectional, which limits conclusions about causality and the temporal sequence of microbiome alterations relative to autoantibody development. These limitations emphasize the need for larger, standardized, and longitudinal studies that explicitly stratify both RF and ACPA to validate our observations and clarify their clinical significance. Another limitation is that we were unable to perform a formal meta-analysis due to the small number of eligible studies and the substantial heterogeneity in study design, sequencing methods, and outcome reporting. This precluded quantitative synthesis and restricted our analysis to a qualitative approach.

## 5 Conclusion

This systematic review demonstrates that gut microbiome alterations in RA differ by serological status, although reported taxa are heterogeneous across studies. The most consistent findings include enrichment of *Collinsella* and *Blautia* and depletion of *Faecalibacteriu*m in seropositive patients, while other associations were observed only in single cohorts. Preclinical ACPA-positive individuals showed distinct microbial profiles, suggesting that dysbiosis may precede clinical disease onset. These results highlight the importance of accounting for RF and ACPA status in microbiome research and support further standardized, large-scale studies to validate candidate taxa and assess their diagnostic and therapeutic potential.

## Data Availability

The original contributions presented in the study are included in the article/**Supplementary Material**. Further inquiries can be directed to the corresponding author.

## Glossary

ACPA: Anti-citrullinated protein antibodies
DMARDs: Disease-modifying anti-rheumatic drugs
BDMARDs: Biologic DMARDs
CsDMARDs: Conventional synthetic DMARDs
NOS: Newcastle–Ottawa Scale
NSAIDs: Nonsteroidal anti-inflammatory drugs
NR: Not reported
RA: Rheumatoid arthritis
RF: Rheumatoid factor
α-diversity: A measure of microbial diversity within a sample
β-diversity: A measure of differences in microbial composition between samples
Biomarker: A measurable indicator of a biological condition or process
Dysbiosis: An imbalance in the composition and function of the gut microbiota
Taxa: Groups of organisms in biological classification
