# Supplementary material for: Gut microbiome differences by serostatus in rheumatoid arthritis: a systematic review
Source: Front Immunol. 2026 Mar 25;17:1722255. doi: 10.3389/fimmu.2026.1722255 (PMC13057437; doi:10.3389/fimmu.2026.1722255)
Supplement: Supplementary file 1 [file Table1.docx]

**Supplementary table 1. Search strategy and data sources**

| **Database** | **Intervention** | **Search terms** | **Results (number of hits)** | **Date and time** |
| --- | --- | --- | --- | --- |
| PubMed | RF/ACPA | ("rheumatoid arthritis"[All Fields] OR "RA"[All Fields]) AND ("gut microbiota"[All Fields] OR "gut microbiome"[All Fields] OR "intestinal microbiota"[All Fields] OR "dysbiosis"[All Fields]) AND ("ACPA"[All Fields] OR "anti-CCP"[All Fields] OR "rheumatoid factor"[All Fields] OR "RF"[All Fields] OR "autoantibodies"[All Fields]) | 89 (6) | 23 July 2024, 06:42:03 |
| Web of science | RF/ACPA | TS=(("rheumatoid arthritis" OR RA OR "autoimmune arthritis") AND ("gut microbiome" OR "gut microbiota" OR "intestinal microbiota" OR dysbiosis OR "gut flora" OR "intestinal flora") AND (ACPA OR "anti-CCP" OR "rheumatoid factor" OR RF OR serostatus OR autoantibodies)) | 73 (6) | 23 July 2025 |
| Scopus | RF/ACPA | TITLE-ABS-KEY ( ( "rheumatoid arthritis" OR RA ) AND ( "gut microbiome" OR "gut microbiota" OR "intestinal microbiota" OR dysbiosis ) AND ( ACPA OR "anti-CCP" OR "rheumatoid factor" OR RF OR serostatus OR autoantibodies ) ) | 167 (8) | 23 July 2025 |
| Cochrane Library | RF/ACPA | ("rheumatoid arthritis" OR "RA") AND ("gut microbiome" OR "gut microbiota" OR "intestinal flora" OR dysbiosis) AND ("rheumatoid factor" OR RF OR "anti-cyclic citrullinated peptide" OR ACPA OR "serological status" OR serostatus) | 9 (0) | 23 July 2025 |
